# Lignin as Polymer Electrolyte Precursor for Stable and Sustainable Potassium Batteries

**DOI:** 10.1002/cssc.202200294

**Published:** 2022-05-18

**Authors:** Sabrina Trano, Francesca Corsini, Giuseppe Pascuzzi, Elisabetta Giove, Lucia Fagiolari, Julia Amici, Carlotta Francia, Stefano Turri, Silvia Bodoardo, Gianmarco Griffini, Federico Bella

**Affiliations:** ^1^ Department of Applied Science and Technology Politecnico di Torino Corso Duca degli Abruzzi 24 10129 Torino Italy; ^2^ Department of Chemistry, Materials and Chemical Engineering “Giulio Natta” Politecnico di Milano Piazza Leonardo da Vinci 32 20133 Milano Italy; ^3^ National Interuniversity Consortium of Material Science and Technology (INSTM) Via Giuseppe Giusti 9 50121 Firenze Italy

**Keywords:** batteries, biomass, lignin, polymer electrolytes, potassium

## Abstract

Potassium batteries show interesting peculiarities as large‐scale energy storage systems and, in this scenario, the formulation of polymer electrolytes obtained from sustainable resources or waste‐derived products represents a milestone activity. In this study, a lignin‐based membrane is designed by crosslinking a pre‐oxidized Kraft lignin matrix with an ethoxylated difunctional oligomer, leading to self‐standing membranes that are able to incorporate solvated potassium salts. The in‐depth electrochemical characterization highlights a wide stability window (up to 4 V) and an ionic conductivity exceeding 10^−3^ S cm^−1^ at ambient temperature. When potassium metal cell prototypes are assembled, the lignin‐based electrolyte attains significant electrochemical performances, with an initial specific capacity of 168 mAh g^−1^ at 0.05 A g^−1^ and an excellent operation for more than 200 cycles, which is an unprecedented outcome for biosourced systems in potassium batteries.

## Graphical abstract

**A lignin‐based membrane** is designed by crosslinking a pre‐oxidized Kraft lignin matrix with an ethoxylated difunctional oligomer, affording self‐standing membranes that incorporate solvated potassium salts and exhibit a wide stability window and ionic conductivity exceeding 10^−3^ S cm^−1^ at ambient temperature. Battery prototypes show an initial specific capacity of 168 mAh g^−1^ at 0.05 A g^−1^ and stability for more than 200 cycles.

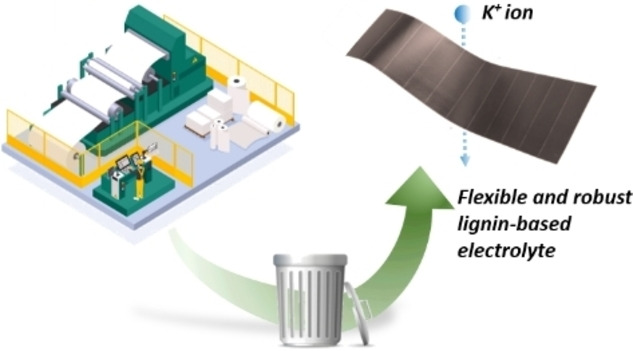

## Introduction

The current growth in the exploitation of renewable energy sources increases the need for efficient energy storage systems, in particular electrochemical devices such as batteries.[^1^, ^2^, ^3^, ^4^, ^5^] At present, lithium‐ion batteries (LIBs), working through the rocking chair mechanism, dominate the market of electrochemical energy storage devices, owing to their superior energy/weight ratio, long cycle life and fast charge‐discharge processes.[^6^, ^7^, ^8^, ^9^, ^10^] However, the scarcity of lithium in the Earth crust (0.0017 wt%) threatens the exploitation of LIBs in large‐scale energy storage systems for storing the electricity produced by photovoltaics and wind plants.[^11^, ^12^, ^13^, ^14^, ^15^]

Sodium and potassium are more abundant alkali metals (2.3 and 1.5 wt% in the Earth crust, respectively) and can potentially replace lithium in rocking chair batteries, at least for the aforementioned stationary applications; indeed, in these cases battery size is not relevant, while the investment cost becomes a primary target.[^16^, ^17^, ^18^] In addition, sodium and potassium can be used in combination with light and cheap aluminum as current collector (instead of copper), since they do not alloy with this metal during the charge‐discharge process.[^19^, ^20^, ^21^] Potassium offers peculiar advantages with respect to sodium. For example, it shows a more negative redox potential (−2.936 V vs. SHE), being rather close to that of lithium (−3.040 V vs. SHE).[^22^, ^23^, ^24^] Also, K^+^ is a weaker Lewis acid with respect to sodium and lithium cations, thus exhibiting a lower solvation (i. e., smaller solvated ions) in the liquid electrolyte.^[25]^ Owing to all these reasons, the research on potassium‐ion batteries (PIBs) has been strongly sustained over the last three years and the scientific community has developed several electrode materials able to reversibly store and release potassium ions.[^26^, ^27^] Even if the electrolyte plays a significant role in battery design and engineering, ruling the charge balance during the operation and being responsible for the establishment of a stable interfacial contact with the electrodes,[^28^, ^29^, ^30^] very few works related to this battery component have been reported in the PIBs framework. From the LIBs field, it is well known that a suitable electrolyte must ensure a proper ion mobility and charge transfer, as well as being safe, (electro)chemically stable and environmentally friendly.[^31^, ^32^, ^33^, ^34^] Nowadays, the best‐known electrolytes for PIBs are liquid‐state systems based on organic solvents that, despite ensuring high conductivity, also exhibit poor safety, high flammability, and solvent evaporation and leakage; moreover, they do not impede the growth of metal dendrites.[^35^, ^36^, ^37^, ^38^] In contrast, despite lower ion conductivity, (quasi‐)solid state electrolytes offer improved safety and stability and can suppress potassium dendrite growth; among them, gel polymer electrolytes (GPEs) offer a higher charge mobility and adapt to the surface of the electrodes, ensuring a better interfacial electrochemical contact.[^39^, ^40^, ^41^, ^42^] While many GPEs have been reported for both LIBs[^39^, ^43^, ^44^] and sodium‐based systems,[^45^, ^46^, ^47^, ^48^] the research on GPEs for PIBs is still in its infancy and has been mainly based on already established oil‐derived polymeric matrices, such as poly(propylene carbonate), poly(ethylene oxide) and poly(methyl methacrylate).[^49^, ^50^, ^51^, ^52^]

In this framework, it should be considered that cells manufacturing at a large scale should pass through low‐impact materials, ideally coming from spent batteries, other wastes or biosourced components. As regards the latter, the wide sector of biomass transformation makes the scientific community aware of the fact that lignocellulose can be considered as a valuable alternative to fossil fuels, owing to its high abundance and rapid regeneration.[^53^, ^54^] Lignocellulose is composed by a variety of polymers and molecules, such as cellulose, hemicellulose, lignin and tannin.^[55]^ Among them, lignin is the most abundant aromatic polymer on Earth and is, at the same time, a waste coming from the paper‐making industry. Currently, the majority of the produced lignin is burnt and its potential use as a functional biopolymer is still underexploited. However, the direct utilization of lignin after a suitable physical/chemical transformation, given the high amount of functional groups it exhibits, represents an interesting strategy to obtain low cost, added value materials. For example, during the last few years, some intriguing lignin‐based components have been developed and used in the broad field of sustainable manufacturing,[^56^, ^57^, ^58^] as well as in more high‐tech applications such as solar cells,^[59]^ supercapacitors,^[60]^ 3D printable inks^[61]^ and CO_2_ capture units.^[62]^

Herein, we present the first biosourced polymer electrolyte system for PIBs. A lignin‐based membrane was designed by crosslinking a pre‐oxidized Kraft lignin matrix (OxL) with poly(ethylene glycol) diglycidyl ether (PEGDGE). Subsequently, the membrane was activated by incorporating a potassium salt solution in its void spaces and subjected to an in‐depth characterization. Such crosslinked system allowed the reduction of the evaporation of the liquid component of the electrolyte, thus increasing the stability and the life cycle of the lab‐scale prototypes, in addition to suppressing the formation of potassium dendrites. The newly conceived GPE showed high ionic conductivity at room temperature, a reversible plating/stripping behavior and, when assembled in potassium/membrane/carbon half‐cells, exhibited an initial specific capacity of 168 mAh g^−1^ at 0.05 A g^−1^ and excellent operation for more than 200 cycles.

## Results and Discussion

### Characterization of OxL/PEGDGE‐based membranes

**Figure 1:**
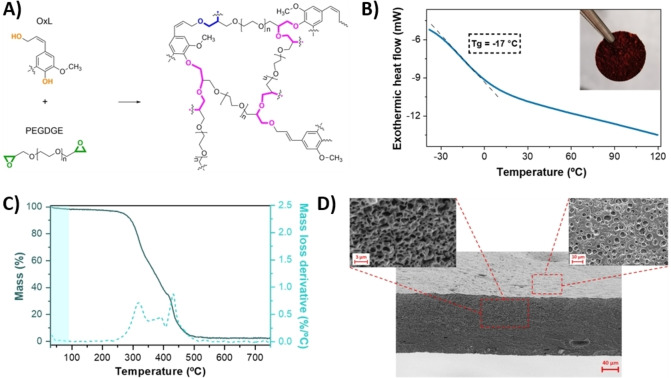
A) PEGDGE‐mediated crosslinking of OxL under alkaline conditions. B) DSC trace of the OxL/PEGDGE‐based membrane. Inset: photographic image of a 20 mm‐diameter OxL/PEGDGE membrane. C) TGA and DTG profiles of the OxL/PEGDGE membrane, where the extent of mass loss in the working temperature window (<100 °C) is highlighted in light blue. D) Cross‐section and top‐view SEM images of a representative cryo‐fractured OxL/PEGDGE membrane, where the internal and surface porous structure is evident.

Based on recent findings from our research groups,^[59]^ the crosslinking reaction between OxL and PEGDGE (Figure 1A) was exploited to produce a bio‐based membrane with sufficiently high molecular mobility and self‐standing capability (*T*_g_=−17 °C, Figure 1B) to be used as GPE in PIBs.

TGA measurements in air were employed to gauge the thermo‐oxidative stability of such lignin‐based membranes when subjected to relevant operating conditions. As shown in Figure 1C, no appreciable mass loss events (<1 %) were recorded within the typical working temperature window of PIBs (<100 °C). Significant weight losses were observed only for temperatures higher than 200 °C (*T*_onset_ ≈240 °C), with major degradation phenomena (*T*_max_) associated with the rupture of α‐ and β‐aryl‐alkyl ether linkages and aliphatic chains in lignin (250–350 °C), ultimately leading to the complete rupture of the macromolecular crosslinked structure (>350 °C) and yielding a residual weight at 750 °C of 2.3 %.[^63^, ^64^] These evidences clearly suggest the excellent thermal stability of such lignin‐based membranes for the target application.

The morphology of the as‐prepared lignin‐based membranes was assessed by SEM on cryo‐fractured samples. As shown in Figure 1D, a densely interconnected internal porous structure was formed upon reaction between OxL and PEGDGE, with small pore size of characteristic dimensions of around 1 μm or smaller. A similar porous structure was also reflected on the surface morphology of the crosslinked materials. Such homogeneously packed three‐dimensional architecture is known to be beneficial for the structural and functional response of the solid membrane as it is expected to provide reduced brittleness, improved compressive strength and enhanced liquid electrolyte retention upon soaking.^[65]^

**Figure 2:**
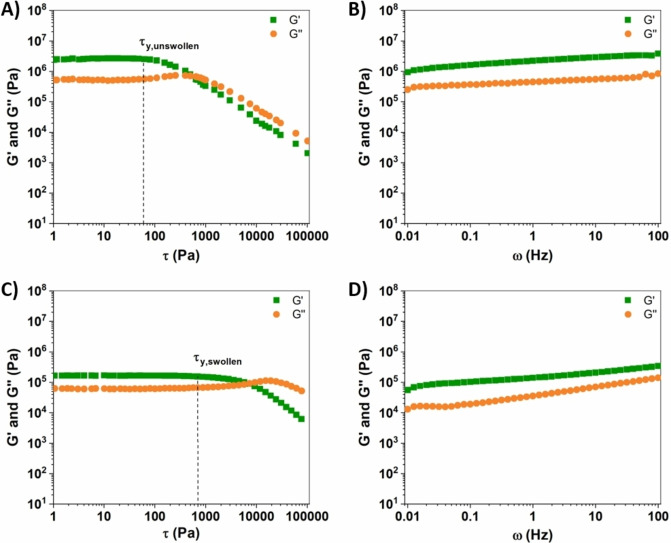
A,C) *G*′ and *G*′′ obtained by dynamic shear stress‐sweep rheological measurements on OxL/PEGDGE‐based membranes: A) unswollen membrane; C) membrane swollen using a 1 : 1 v/v EC/DEC solution. The *τ*_y_ value is indicated. B,D) *G*′ and *G*′′ obtained by dynamic frequency‐sweep rheological measurements on OxL/PEGDGE‐based membranes: B) unswollen membrane; D) membrane swollen using a 1 : 1 v/v EC/DEC solution.

Another important parameter correlated to the structural features of the lignin‐based membranes and affecting their swelling ability is represented by their crosslinking density (ν). Indeed, ν is known to govern the mobility of the three‐dimensional polymer network, its mechanical response and ultimately the solvent diffusion process within the membrane structure. Based on these considerations, ν of unswollen membranes could be estimated according to the molecular theory of rubber elasticity [see Experimental Section, Eq. (1)] from the $G_{R}^{'}$ value as measured by dynamic rheology tests in frequency sweep configuration within the linear viscoelastic region (LVR) (Figure 2A,B). Accordingly, a ν value of 1.16 ⋅ 10^−3^ mol cm^−3^ was found for such lignin‐based membranes, in line with other similar lignin‐based crosslinked gel systems recently reported for different applications.[^66^, ^67^]

To examine the structural stability and viscoelastic response of the OxL/PEGDGE‐based membrane materials in the swollen state, the dependence of the shear storage modulus (*G*′) and the shear loss modulus (*G*′′) on the shear stress (*τ*) and on the frequency (*ω*) was investigated by means of rheological tests carried out after soaking the membrane in a 1 : 1 v/v EC/DEC solution to reach swelling equilibrium. When compared to the unswollen counterpart (Figure 2A), swollen membranes were found to be characterized by lower *G*′ and *G*′′ values as a consequence of the increased end‐to‐end distance of the chains and of the expansion of the pore structure through solvent intercalation.^[68]^ Moreover, the LVR was greatly extended upon membrane swelling (Figure 2C). In particular, the yield stress (*τ*_y_) limiting the LVR was found to increase from around 60 MPa for the unswollen material (Figure 2A) to around 700 MPa for the swollen gel (Figure 2C). This behavior evidences the beneficial effect of the presence of solvent entrapped within the porous structure of the membrane, shifting the onset of irreversible plastic deformation in the swollen membranes to significantly higher stress values. This is a key aspect in the view of the final application as GPE in PIBs, as a higher mechanical strength (and extended elastic response) allows to better withstand volume changes during charge‐discharge cycles and during cell assembly processes, to better accommodate dendrite growth and potentially to be able to use thinner and more conductive membranes.

Finally, dynamic rheology frequency sweep tests (Figure 2B,D) demonstrated that, for both unswollen and swollen membranes, *G*′ is higher than *G*′′ in the whole frequency range investigated (0.01–100 Hz). This finding indicates that the elastic response of these membrane materials is always predominant over the viscous one, thus proving their solid‐like behavior, their effective crosslinked nature and their excellent mechanical rigidity.^[63]^ In addition, a slight increase in both *G*′ and *G*′′ is observed for higher frequency (*viz*., lower relaxation times) associated to a progressively increased membrane rigidity, in line with reports on analogous crosslinked gel systems.^[69]^

### Electrochemical characterization of OxL/PEGDGE‐based membranes as GPE

**Figure 3:**
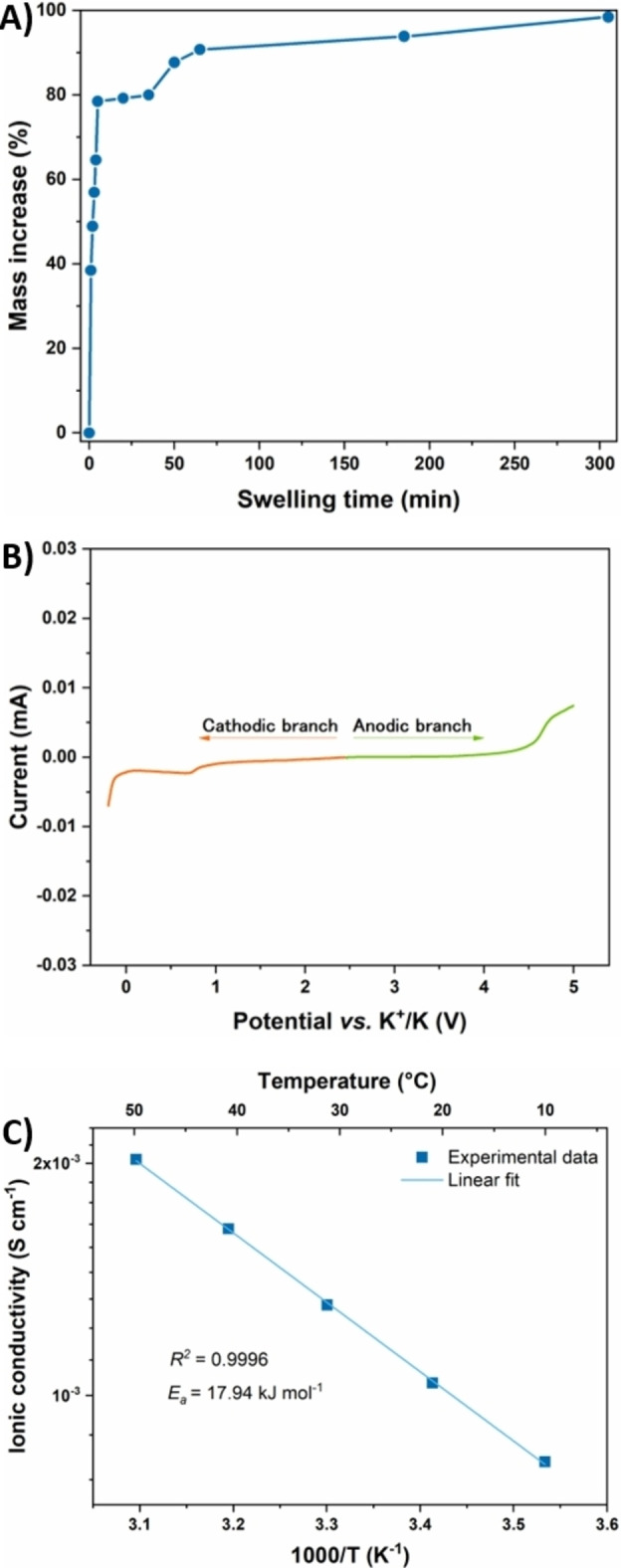
A) Swelling curve of the OxL/PEGDGE membrane immersed in a liquid electrolyte based on 0.80 m KPF_6_ in 1 : 1 v/v EC/DEC. B) LSV of a K/GPE/SS cell, studied at room temperature, between −0.2 and 5 V at a scanning rate of 0.1 mV s^−1^. C) *σ* values of the lignin‐based GPE as a function of temperature (from 10 °C to 50 °C), fitted with the Arrhenius’ model.

The success of a newly conceived GPE primarily depends on its ability to uptake a suitable amount of liquid electrolyte, which is responsible for the membrane activation process through the insertion of solvated potassium ions within the macromolecular matrix. The swelling is considered efficient if the liquid electrolyte remains entrapped in the crosslinked polymer network and if it leads to high ionic conductivity values, while keeping at the same time the mechanical properties of a self‐standing membrane. Figure 3A shows the mass increase profile over the swelling time, highlighting that the membrane can efficiently uptake the liquid electrolyte within its interconnected porous structure, yielding an 80 % mass increase already after 5 min of immersion, as a result of the favorable ν. In terms of liquid electrolyte volumetric uptake, a membrane with a diameter of 18 mm and a thickness of 150 μm incorporated 61 μL of solution.

Since one of the main critical factors affecting the operation of PIB systems is represented by the high reactivity of potassium metal, a linear sweep voltammetry (LSV) experiment was mandatory to examine both anodic and cathodic stabilities of the swollen OxL/PEGDGE membranes when interfaced with potassium at different potential values. The curve shown in Figure 3B reveals a wider stability window than that required to meet the practical applications of a PIB (i. e., below 4 V). Only a very small parasitic current, in the order of 10^−6^ A, was recorded at ≈0.8 V, which could be attributed to a weak reactivity between −OH groups present in the lignin matrix and the potassium metal surface. However, being the magnitude of this signal commonly considered insignificant in the polymer electrolytes field,[^70^, ^71^, ^72^] the proposed electrolyte demonstrated its full operability in the electrochemical working conditions typical of PIBs.

Ionic conductivity (*σ*) values were investigated between 10 and 50 °C (Figure 3C). Experimental values were perfectly fitted with the Arrhenius’ law, with a linear increase of *σ* when raising temperature. Interestingly, a rather low difference was noticeable between the *σ* value measured at ambient temperature and that recorded at 50 °C, being both of them in the order of 10^−3^ S cm^−1^, which already represents a very good value for a GPE to be used in secondary PIBs.^[31]^ Such a noteworthy result was attributed to the homogenous three‐dimensionally crosslinked architecture of the solid electrolyte matrix, showing an efficient liquid electrolyte entrapping ability and a reduced brittleness, as also corroborated by rheological analysis.

**Figure 4:**
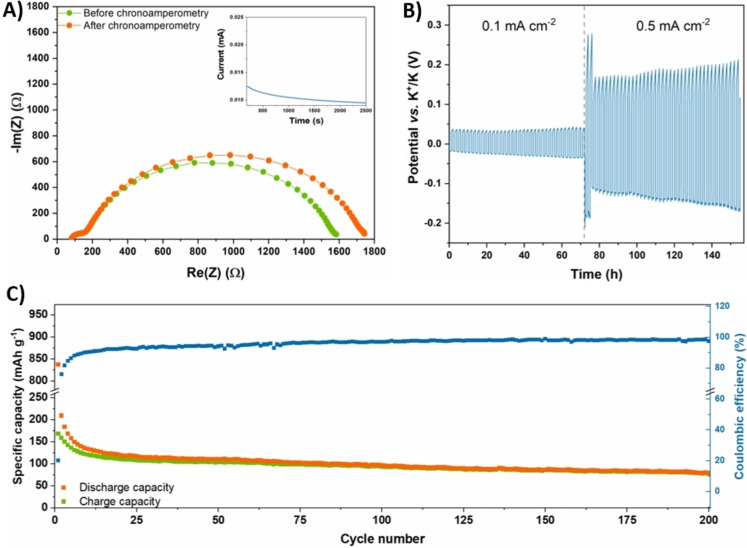
A) EIS spectra of symmetric K/GPE/K cell before and after polarization. The current profile response to the DC voltage step is shown in the inset. B) Potential vs. test time of potassium stripping and plating carried out in a symmetrical K/GPE/K cell at 0.1 mA cm^−2^ and 0.5 mA cm^−2^ (see the Supporting Information, Figure S1, which shows a zoomed portion of the experimental curve). C) Cycling performances of a Super‐P/GPE/K half‐cell tested at a specific current of 0.05 A g^−1^ for 200 cycles.

A homogenous and efficient cation transference through the solid electrolyte is vital for the cyclability, the safety and the lifespan of a battery. The transference number ($t_{K^{+}}$ ) is a significant figure of merit that quantifies this electrolyte feature, ranging between 0 and 0.5 if only positive charge motion is considered. The proposed lignin‐based GPE was found to display a $t_{K^{+}}$ as high as 0.24 when tested according to the method proposed by Evans et al. (Figure 4A).^[73]^ Such a value represents a very encouraging result when considering that PIBs are an emerging electrochemical energy storage technology and the majority of the studies regarding the development of quasi‐solid/solid electrolytes for PIBs do not report $t_{K^{+}}$ data.

Galvanostatic cycling profiles for the symmetrical K/GPE/K cell are really useful to describe the electrolyte/potassium interface behavior. Keeping in mind that positive voltages refer to potassium stripping, whereas negative ones refer to potassium plating, Figure 4B shows the results of galvanostatic cycling in two distinct current density regimes, namely 0.1 mA cm^−2^ and 0.5 mA cm^−2^. In the first regime (0.1 mA cm^−2^), the voltage profile indicates that overpotentials are encouragingly low, likely suggesting an even deposition of potassium. The corresponding electrochemical signals are very symmetric, proving equal performances on both K/GPE interfaces, thanks to the homogenous crosslinked polymeric network. The obtained values were maintained nearly constant for over 36 cycles, indicating a uniform and stable solid electrolyte interphase (SEI) formation. When the system was subjected to a more challenging current density value (0.5 mA cm^−2^), a higher overpotential for the first cycles was required, suggesting some changes in the interfacial structure in order to accommodate an increased cations motion. However, a subsequent decrease in overpotential was observed after these first cycles at 0.5 mA cm^−2^, proving that a stabilized interface between the potassium layer and the GPE was achieved. The improvement of the interfacial structure was also proved by the decrease of the internal resistance at the K/GPE interfaces, as quantified by the semicircle diameter of symmetrical cell in EIS spectra. Indeed, Figure S2 shows that the diameter dropped from 1900 to 500 Ω and, again, increased a bit (800 Ω) at a higher current density. No failure of the cell was detected in 77 cycles (154 h), validating how the high mechanical strength of the lignin‐based membrane, associated with its elastic behavior, can counteract dendrite growth, thus ensuring excellent device cycling stability. Different results were obtained by the commercial separator Celgard® 2500, which we tested ‐ for the sake of comparison ‐ under identical conditions. Several overvoltage peaks indicate the nucleation of potassium dendrites; furthermore, the voltage profile follows a step‐like behavior, suggesting that no plating, neither stripping of potassium ions are occurring. Even if it does not seem to reach short circuit conditions, as noticeable in Figure S3, this behavior may suggest the proximity of dendrites on the two interfaces, also proved by the huge decrease of the internal resistance detected between the first and second EIS measurements, that is, before the galvanostatic polarization and after 36 cycles, respectively. Indeed, an internal resistance to the charge transfer as low as 6 Ω indicates that no ions are being actually transferred (Figure S4).

**Figure 5:**
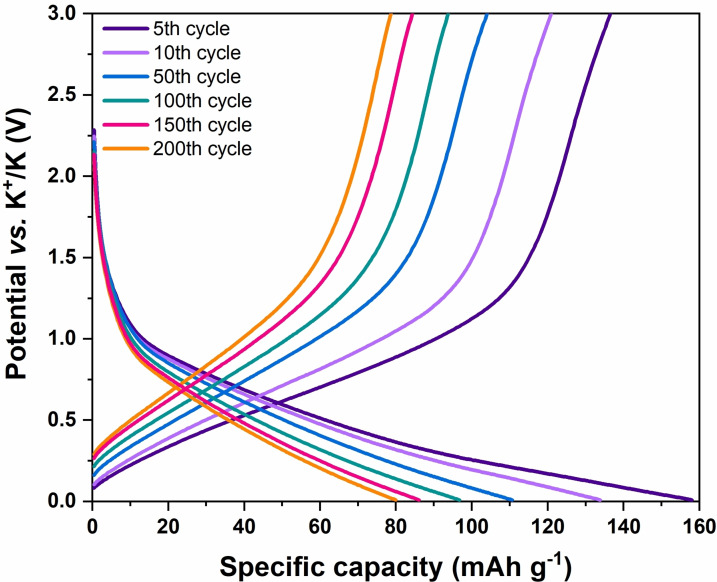
Representative charge‐discharge profiles at the 5^th^, 10^th^, 50^th^, 100^th^, 150^th^, and 200^th^ cycle for a Super‐P/GPE/K half‐cell cycled at 0.05 A g^−1^.

After assessing the compatibility of the biobased GPE in PIBs, its electrochemical energy storage performance was investigated in K/GPE/Super‐P half‐cells carrying out the rate performance (Figures S5 and S6), the results of which denote the GPE ability to tolerate increasing currents. A long‐cycling performance at a constant current density of 0.05 A g^−1^_active mass_ was performed too; the resulting specific capacity values vs. cycle number are shown in Figure 4C. A rather low Coulombic efficiency (CE) was detected during the very first cycles. This behavior was ascribed to the carbon black Super‐P electrode, which is widely known to have different chemisorbed species (i. e., hydroxy, carboxyl and carbonyl groups) that irreversibly react with potassium ions during the first discharges;^[74]^ also, an initially low CE has been previously attributed to DEC consumption during SEI layer formation.^[75]^ As a matter of fact, during this transient regime, charge capacities are almost constant, while the discharge ones tend to decrease reaching the charging values. Once the SEI layer was formed and the carbon black interface became stable, a constantly increasing CE was obtained, achieving a plateau value of 98 %. At the same time, a very slow capacity fading and a considerable capacity retention was also observed for this system. Indeed, the coin cell was able to retain 64.18 % of the charge capacity recorded at the 10^th^ cycle after as many as 200 cycles. Remarkably, this represents a capacity decay rate as low as 0.18 % per cycle with respect to the 10^th^ cycle, following a trend that is found to further decrease upon cycling. Indeed, as shown by the charge and discharge profiles reported in Figure 5 (5^th^, 10^th^, 50^th^, 100^th^, 150^th^ and 200^th^ cycles were taken as representative), the shift in specific capacity between the curves constantly decreases at increasing cycle number, demonstrating a clear reduction of the capacity fading with operational lifetime. Furthermore, the half‐cell was able to cycle for over 500 cycles with good performances (Figure S7) and its Super P electrode morphology was studied by FESEM micrographs and compared with a fresh one. The latter (Figure S8A) shows a porous morphology, similar to reported findings.^[76]^ The morphology was maintained after the charge‐discharge cycles (Figure S8B). Some traces of the electrolyte salt are evident as well.

To our knowledge, no other studies on biosourced GPEs for PIBs have shown similar performance to date. As inferred from the electrochemical results, and above all from the conclusions drawn from Figure 4B, we hypothesize that the formation of a stable and homogeneous SEI layer, favored by the presence of the lignin‐based GPE, can be considered as the principal reason for the encouraging cycling stability observed for these PIBs prototypes. We are currently setting up an in‐depth analysis of the SEI layers formed when potassium‐based electrolytes (even laden with specific additivities, such as carbonate compounds) are introduced in different polymeric matrices, and this will constitute the matter of a forthcoming publication.

## Conclusions

A lignin‐based polymer electrolyte has been proposed for the fabrication of potassium metal batteries. Among the most intriguing electrochemical outcomes, the proposed quasi‐solid system demonstrated a stability window up to 4 V and an ionic conductivity exceeding 10^−3^ S cm^−1^ at ambient temperature. Lab‐scale battery prototypes showed an initial specific capacity of 168 mAh g^−1^ at 0.05 A g^−1^ and excellent operation for more than 200 cycles, placing this lignin‐based separator among the best‐performing biosourced systems in the field of potassium batteries.

## Experimental Section

### Materials

The softwood Kraft lignin used in this work (Indulin AT) was supplied by Ingevity. PEGDGE (*M*_n_ 500 g mol^−1^), ferrous chloride tetrahydrate (FeCl_2_ ⋅ 4H_2_O, analytical grade), hydrogen peroxide (H_2_O_2_, 30 wt% in H_2_O), sulfuric acid (H_2_SO_4_, ACS reagent, 95.0–98.0 %), sodium hydroxide (NaOH, ACS reagent, ≥97.0 %, pellets), potassium (cubes in mineral oil, 99.5 % trace metals basis), potassium hexafluorophosphate (KPF_6_, 99.5 % trace metals basis) and N‐methyl‐2‐pyrrolidone (NMP) were provided by Merck. Ethylene carbonate (EC) and diethyl carbonate (DEC) were bought from Solvionic, battery grade. Carbon black Super‐P and poly(vinylidene fluoride) (PVDF) were purchased from Alfa Aesar. All chemicals were used without any further purification.

### Membrane preparation: lignin oxidation and crosslinking with PEGDGE

Oxidized lignin (OxL) in the form of dry brownish powder was obtained via the Fenton pathway using FeCl_2_ ⋅ 4H_2_O as catalyst and H_2_O_2_ as oxidizing agent, following a recently reported procedure.^[59]^ This pre‐oxidation step was performed in order to increase lignin reactivity toward PEGDGE and eventually afford mechanically stable lignin‐based membranes. Once OxL was completely dried, it was solubilized in a 3.3 m aqueous NaOH solution (4 mL_solution_ g^−1^_OxL_) under magnetic stirring for 48 h at 50 °C. Then, the solution was cooled down to room temperature and a given amount of PEGDGE with respect to OxL (0.5 g_OxL_ g^−1^_PEGDGE_, corresponding to a OH/epoxy equivalent ratio of 0.88 mol/mol) was added under magnetic stirring to achieve crosslinking of OxL with PEGDGE. Finally, the as‐prepared OxL‐PEGDGE formulation was deposited in a Petri dish on a cellulose acetate film used to facilitate membrane peeling and allowed to rest for 24 h at room temperature, prior to drying in a vacuum oven at 50 °C for 24 h. The membranes were then washed with a 0.2 m aqueous H_2_SO_4_ solution to neutralize the excess of NaOH. After 24 h of drying under vacuum, membranes with a thickness in the range of 100–150 μm were obtained.

### Physicochemical characterization

The glass transition temperature (*T*_g_) of OxL/PEGDGE‐based membranes was measured using a DSC 823e Mettler‐Toledo differential scanning calorimeter (DSC). Approximately 7 mg of the sample were hermetically sealed in an aluminum pan and subjected to two heating and cooling cycles, from −40 to 120 °C, at a rate of 20 °C min^−1^.

Thermogravimetric analysis (TGA) of the OxL/PEGDGE‐based membranes was performed using a TA Instruments Q500 thermogravimetric analyzer. Measurements were carried out in air from 25 to 800 °C, with a constant heating rate of 10 °C min^−1^ and using approximately 15 mg of sample placed in a ceramic crucible. Using the mass loss and mass loss derivative (first‐order thermal derivative, DTG) curves, the degradation onset temperature (*T*_onset_), the temperature at maximum degradation rate (*T*_max_) and the sample residual weight were evaluated.

Dynamic rheological properties of the OxL/PEGDGE‐based membranes both in the swollen (with a 1 : 1 v/v EC/DEC solution) and in the unswollen state were measured using an Discovery DHR2 stress‐controlled rheometer (TA Instruments) equipped with a 20 mm diameter stainless steel (SS) plate and a Peltier‐plate temperature control (parallel‐plate geometry). The linear viscoelastic region (LVR) was measured by varying the shear stress amplitude from 1 to 10^5^ Pa at a constant frequency of 10 Hz. For dynamic shear tests, the frequency was varied between 0.01 and 100 Hz at a fixed shear stress amplitude (10 Pa) to ensure measurements were conducted within the LVR. All experiments were carried out at a constant temperature of 25 °C and applying an active normal force on the specimen (6 n for unswollen samples and 4 n for swollen ones) for the entire duration of the measurements. The value of the shear storage modulus in the rubbery plateau ($G_{R}^{{}^{'}})$ , measured by dynamic rheology frequency‐sweep tests and stress‐sweep tests was used to calculate the crosslinking density (ν; the number of moles of crosslinking sites per unit volume, expressed in mol cm^−3^), according to Equation (1), which is derived from the molecular theory of rubber elasticity:^[77]^

(1) $$\nu =\frac{G_{R}^{{}^{'}}}{\;R\cdot T_{C}\;}$$

where *R* is the gas constant (8.314 J mol^−1^ K^−1^) and *T*_C_ is the absolute temperature (298 K).

Scanning electron microscopy (SEM) analysis in cross section were performed on gold sputtered samples using a Zeiss EVO 50 scanning electron microscope.

### Electrochemical characterization

With the purpose of assessing the application of OxL/PEGDGE‐based membranes in PIBs, a series of electrochemical experiments were carried out. Membrane activation was achieved by swelling in a standard liquid electrolyte (0.80 m KPF_6_ in 1 : 1 v/v EC/DEC), which took place at room temperature for 15 min. As a result, each activated membrane behaved like a GPE, showing mechanical performances comparable to those of a solid‐state electrolyte, but with a lower interfacial resistance when faced with electrochemical cell electrodes.

The ability of these membranes to uptake the liquid electrolyte was expressed by the electrolyte uptake ratio (EUR) coefficient, measured by soaking each polymer membrane sample in the liquid electrolyte at room temperature.^[78]^ The experiment was carried out by weighting the swollen sample every minute five times; then, the process was repeated every 15 min four times and, to conclude, every 2 h twice. The EUR coefficient was obtained by using Equation 2:

(2) $$EUR=\frac{m_{fin}-m_{in}}{m_{in}}\cdot 100\;$$

where *m*_in_ is the initial dry mass of the sample and *m*_fin_ is the weight obtained after each swelling step.

A linear sweep voltammetry (LSV) experiment was conducted at room temperature using two ECC‐Std electrochemical cells (EL‐CELL GmbH), with the aim of determining the electrochemical anodic and cathodic stability limits of the lignin‐based membranes. The electrochemical cells were assembled in an argon‐filled glovebox, using a potassium foil (as both reference electrode and current collector), the GPE and a SS plate (as current collector and working electrode). A VSP‐3e potentiostat (BioLogic Sciences Instruments) was used to record cathodic and anodic currents coming from the two cells. The first one was tested from its open‐circuit voltage (OCV) down to −0.2 V at a scanning rate of 0.1 mV s^−1^, leading to the cathodic current profile. The anodic branch of the LSV data was provided by the second cell, which was subjected to a potential scan from its OCV to 5 V.

A swollen sample sandwiched between two SS plates of an ECC‐Std electrochemical cell was kept into a digital controlled climatic chamber (model MK53 E2.1 by BINDER GmbH) in order to determine the ionic conductivity (*σ*) values at different temperatures. This experiment was carried out by decreasing the temperature from 50 to 10 °C, 10 °C per step. After 1 h of rest at each temperature, an electrochemical impedance spectroscopy (EIS) measurement was performed, obtaining the electrolyte resistance (*R*_b_) value from the intercept at high frequency, needed to calculate *σ* through Equation 3:

(3) $$\sigma =\frac{L}{R_{b}\cdot S}$$

where *L* is the sample thickness and *S* its surface area.

As regards K^+^ transference number ($t_{K^{+}}$ ) measurement, a symmetrical ECC‐Std electrochemical cell, consisting of the GPE sandwiched between two potassium foils, was subjected to a polarization voltage (ΔV) of 20 mV for 2500 s and the initial (*I*_0_) and steady state (*I*_s_ *)* currents were measured. Before and after such chronoamperometry test, the initial (*R*_0_) and steady state (*R*_s_) charge transfer resistances were acquired by using EIS. Then, $t_{K^{+}}$ was obtained according to the Bruce and Vincent's equation [Eq. 4]:

(4) $$t_{K^{+}}=\frac{I_{s}\left(\Delta V-I_{0}R_{0}\right)}{I_{0}\left(\Delta V-I_{s}R_{s}\right)}$$

Several galvanostatic charge and discharge measurements were carried out to gauge the polymer electrolyte response to plating and stripping experiments. A symmetrical ECC‐Std electrochemical cell was subjected to a current density of 0.1 mA cm^−2^ for 1 h for 36 cycles, then the current density was raised up to 0.5 mA cm^−2^ for 41 cycles. The same test was carried out on the commercial separator Celgard® 2500, after being soaked in 0.80 m KPF_6_ in 1 : 1 v/v EC/DEC and sandwiched between two potassium electrodes in a ECC‐Std cell. The electrochemical workstation used to carry out these experiments was a battery testing equipment by Arbin Instruments. EIS measurements were performed by a VSP‐3e potentiostat.

The performance assessment of the newly proposed polymer electrolyte when assembled in lab‐scale PIB prototypes was carried out fabricating LIR2032 coin cells in half‐cell architecture (i. e., carbon black Super‐P onto copper foil/GPE/potassium). Cells were tested between 0.01 and 3 V, by means of the previously mentioned battery testing equipment. Two experimental procedures were adopted: i) a rate performance at the specific current values of 0.05, 0.1, 0.2, 0.4, 0.8, 1 and 2.5 A g^−1^_active mass_ (5 cycles each), followed by 5 more cycles at 0.05 A g^−1^_active mass_; ii) a long‐cycling performance at a constant specific current of 0.05 A g^−1^_active mass_. The cathode was prepared by a formulation consisting of carbon black Super‐P 80 wt% and PVDF 20 wt% in NMP. Ball‐milling (MM 400 Retsch) was used at 30 Hz for 15 min, to uniformly mix the precursors. Then, the resulting homogeneous slurry was deposited onto a Cu‐foil with the doctor blade and an automatic film applicator (TQC Sheen B.V.) set at 50 mm s^−1^ speed. The slurry was cast at a nominal thickness of 200 μm and dried in air at 35 °C overnight. Electrode disks (15 mm of diameter and 1.76 cm^2^ of geometric area) were further dried in a vacuum furnace at 120 °C for 4 h, prior to be brought in an argon filled glove box (<0.5 ppm O_2_, <0.5 ppm H_2_O). The active mass loading of each disk was approximately 0.81 mg cm^−2^. Field emission scanning electron microscopy (FESEM) images were taken for a fresh electrode and a cycled one. The fresh electrode was directly put in a sample holder, while the cycled electrode was recovered from a cell and washed with 1 : 1 v/v EC/DEC. FESEM measurements were carried out with a Zeiss SUPRA 40 equipment with Gemini column and Schottky field emission tip.

## Conflict of interest

The authors declare no conflict of interest.

1

## Supporting information

As a service to our authors and readers, this journal provides supporting information supplied by the authors. Such materials are peer reviewed and may be re‐organized for online delivery, but are not copy‐edited or typeset. Technical support issues arising from supporting information (other than missing files) should be addressed to the authors.

Supporting Information

## Data Availability

The data that support the findings of this study are available from the corresponding author upon reasonable request.
