# Supplementary material for: Lignin as Polymer Electrolyte Precursor for Stable and Sustainable Potassium Batteries
Source: ChemSusChem. 2022 May 18;15(12):e202200294. doi: 10.1002/cssc.202200294 (PMC9322549; doi:10.1002/cssc.202200294)
Supplement: Supplementary file 1 — Supporting Information [file CSSC-15-0-s001.pdf]

ChemSusChem

Supporting Information

Lignin as Polymer Electrolyte Precursor for Stable and Sustainable Potassium Batteries

Sabrina Trano, Francesca Corsini, Giuseppe Pascuzzi, Elisabetta Giove, Lucia Fagiolari, Julia Amici, Carlotta Francia, Stefano Turri, Silvia Bodoardo, Gianmarco Griffini,* and Federico Bella*This publication is part of a Special Collection highlighting "The Latest Research from our Board Members". Please visit the Special Collection at .© 2022 The Authors. ChemSusChem published by Wiley-VCH GmbH. This is an open access article under the terms of the Creative Commons Attribution License, which permits use, distribution and reproduction in any medium, provided the original work is properly cited.

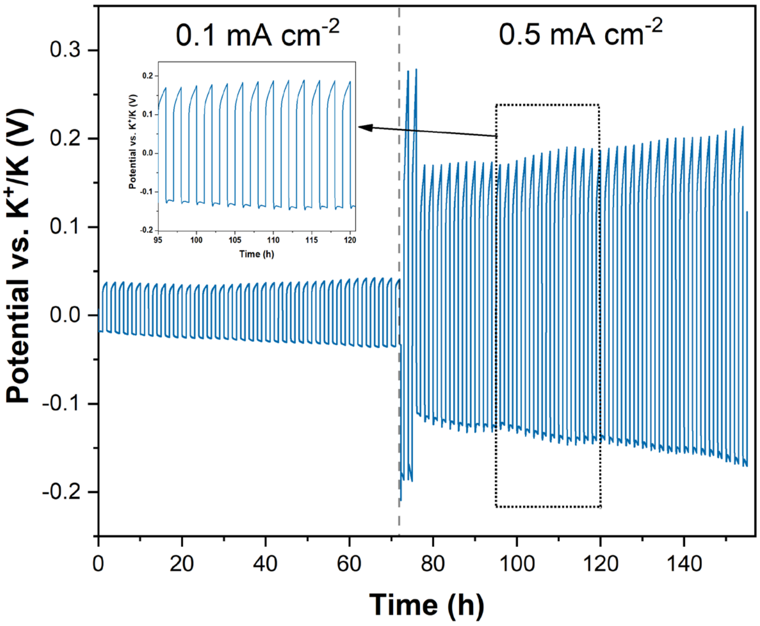

Figure S1 Potential *vs.* test time of potassium plating and stripping carried out in a symmetrical K/GPE/K cell at 0.1 mA cm^{-2} and 0.5 mA cm^{-2}. The inset illustrates the voltage profile of the plating and stripping test, to be compared with the Celgard® 2500 data shown in Figure S3.

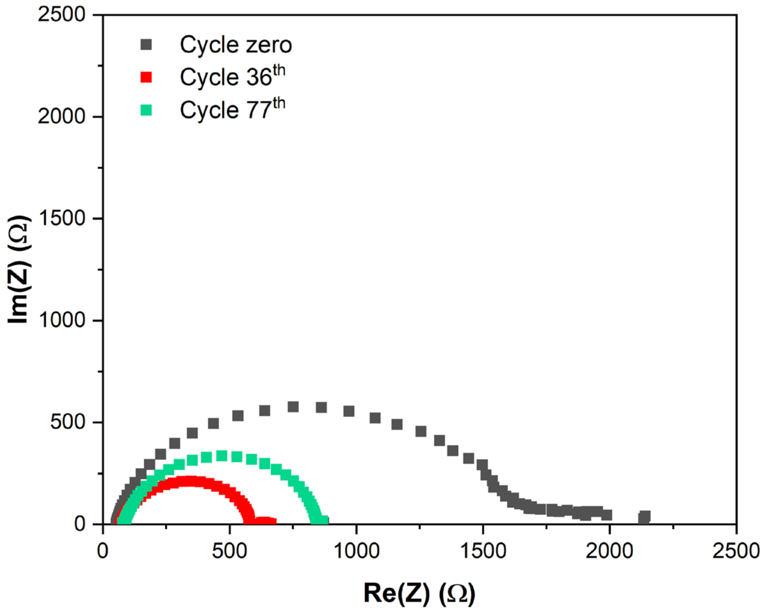

Figure S2 EIS spectra of a symmetrical K/GPE/K cell before the plating and stripping test (in black), after 36 cycles at 0.1 mA cm^{-2} (in red) and after 41 cycle at 0.5 mA cm^{-2} (in green).

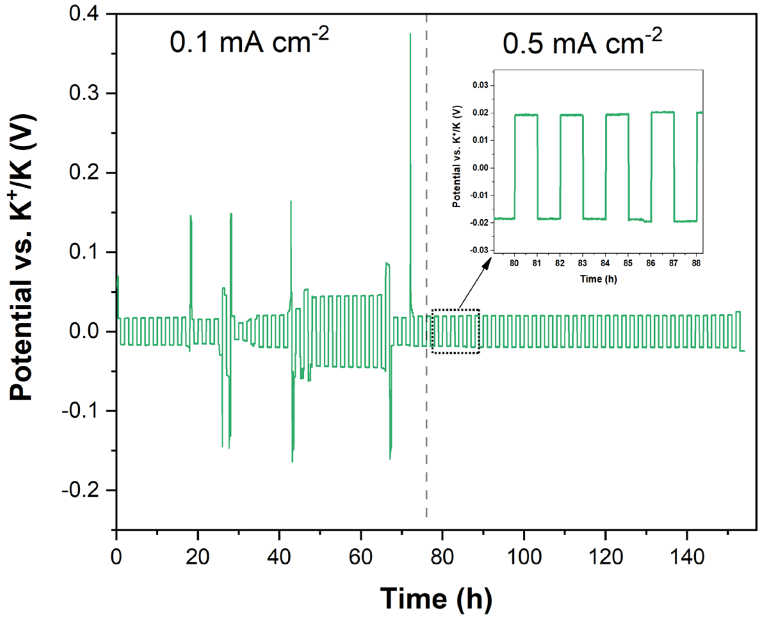

Figure S3 Plating and stripping analysis at two increasing current densities, carried out on the symmetric cell K/Celgard® 2500 swollen in KPF_6 0.80 M in EC:DEC/K. In the inset, the step-like voltage profile is shown.

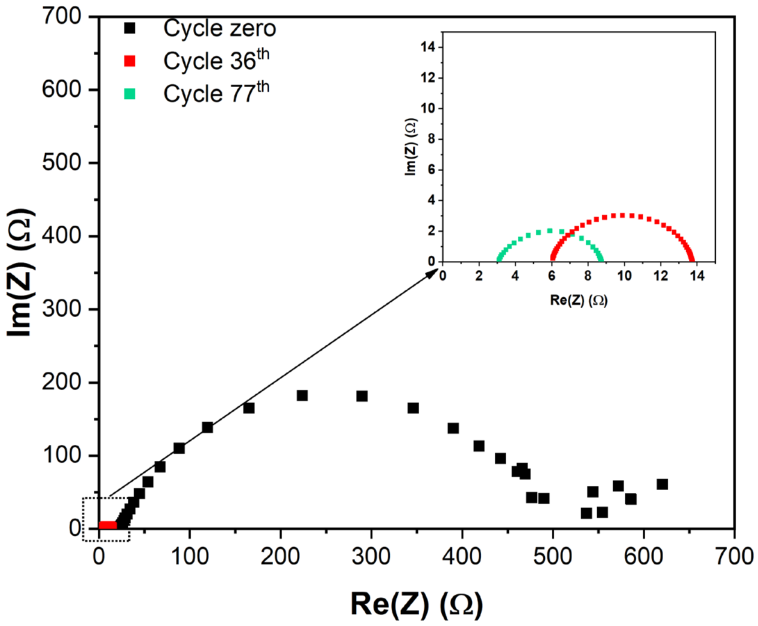

Figure S4 EIS spectra of a symmetric cell fabricated with a Celgard® 2500 separator swollen with KPF_6 0.80 M in EC:DEC. Black symbols refer to data collected before testing, red symbols indicate data recorded after 36 cycles, green symbols regard the measurement taken at the 77th cycle. In the inset, the spectra show internal resistance values equal to 8 Ω and 6 Ω, with respect to the initial resistance of about 450 Ω.

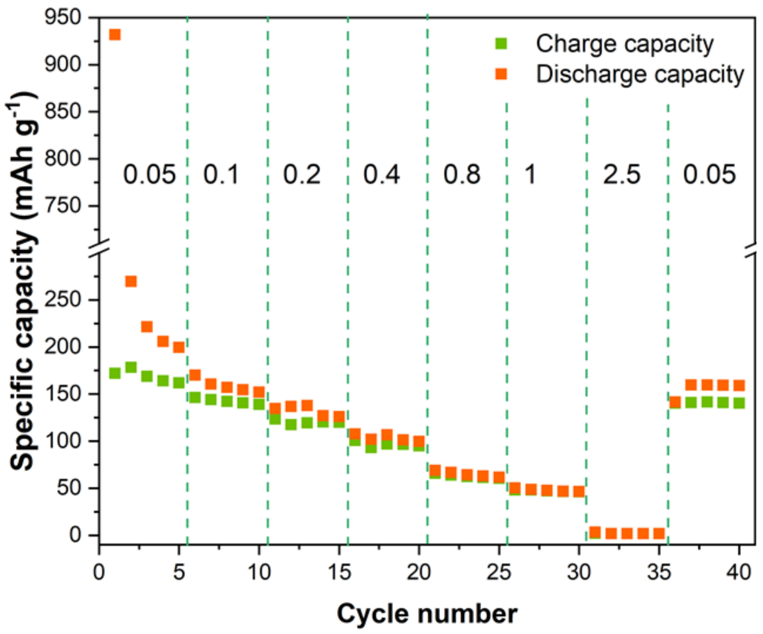

Figure S5 Rate performance of the Super-P/GPE/K half-cell at different specific current values. Numbers between the dashed lines indicate the specific current (A g^{-1}) applied for 5 cycles.

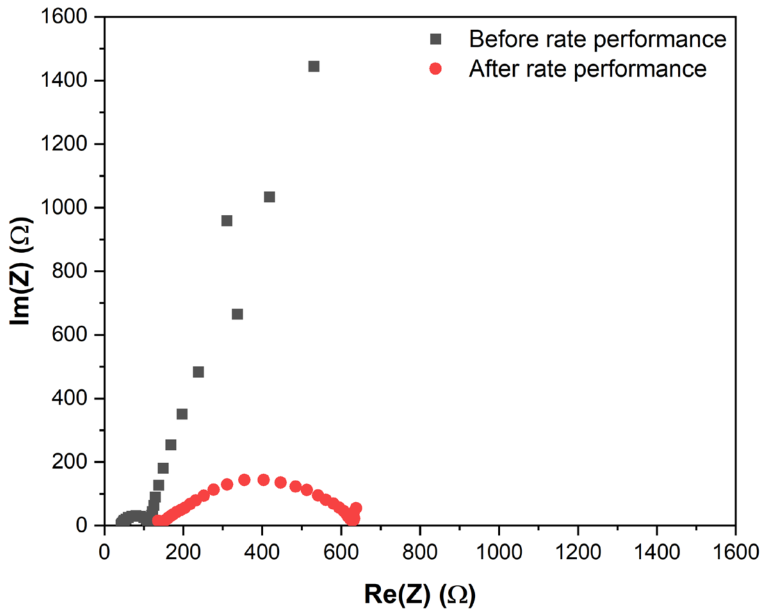

Figure S6 EIS measurements of the Super-P/GPE/K half-cell before and after the rate performance test.

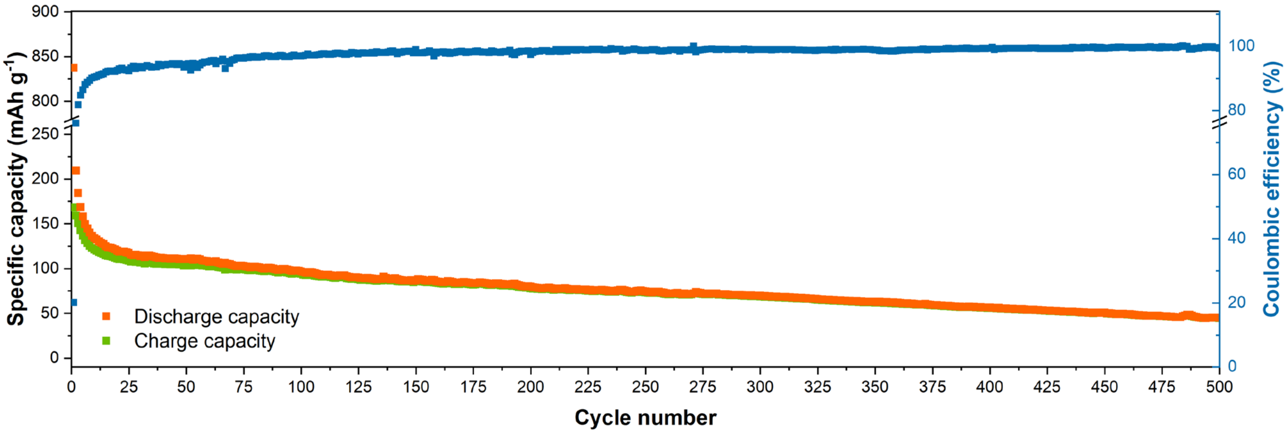

Figure S7 Galvanostatic cycling at constant current of 0.05 A g^{-1} of the Super-P/GPE/K half-cell for 500 cycles.

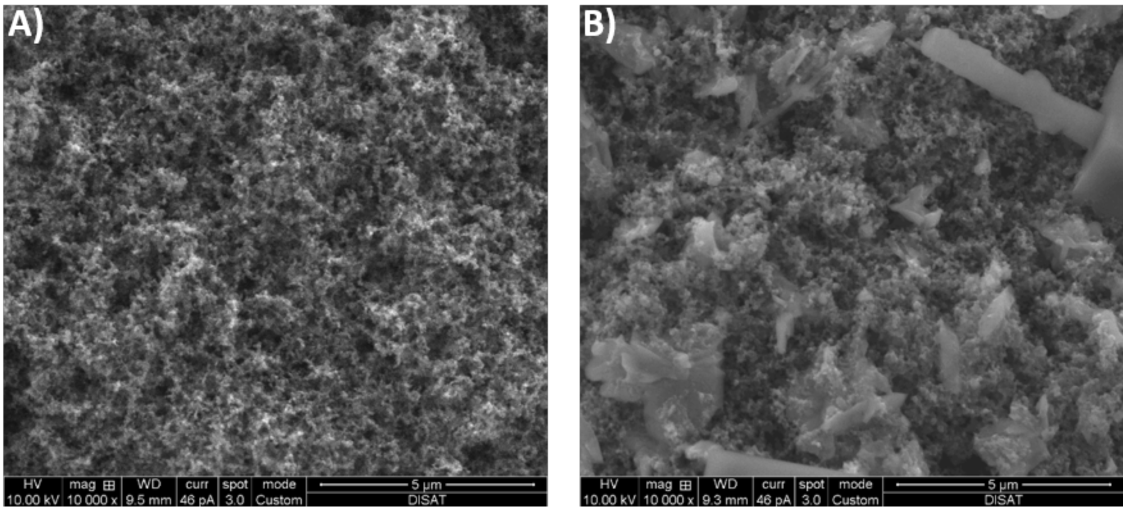

Figure S8 SEM images of A) fresh and B) cycled Super P electrodes.
